# Localization of Bacterial Communities within Gut Compartments across *Cephalotes* Turtle Ants

**DOI:** 10.1128/AEM.02803-20

**Published:** 2021-03-26

**Authors:** Peter J. Flynn, Catherine L. D’Amelio, Jon G. Sanders, Jacob A. Russell, Corrie S. Moreau

**Affiliations:** a University of Chicago, Committee on Evolutionary Biology, Chicago, Illinois, USA; b Drexel University, Department of Biodiversity, Earth and Environmental Science, Philadelphia, Pennsylvania, USA; c Cornell University, Department of Ecology and Evolutionary Biology, Ithaca, New York, USA; d Cornell University, Department of Entomology, Ithaca, New York, USA; Chinese Academy of Sciences

**Keywords:** gut microbes, turtle ants, localization, host-microbe, symbionts

## Abstract

Microbial communities within the animal digestive tract often provide important functions for their hosts. The composition of eukaryotes’ gut bacteria can be shaped by host diet, vertical bacterial transmission, and physiological variation within the digestive tract. In several ant taxa, recent findings have demonstrated that nitrogen provisioning by symbiotic bacteria makes up for deficiencies in herbivorous diets. Using 16S rRNA amplicon sequencing and quantitative PCR, this study examined bacterial communities at a fine scale across one such animal group, the turtle ant genus *Cephalotes*. We analyzed the composition and colonization density across four portions of the digestive tract to understand how bacterial diversity is structured across gut compartments, potentially allowing for specific metabolic functions of benefit to the host. In addition, we aimed to understand if caste differentiation or host relatedness influences the gut bacterial communities of *Cephalotes* ants. Microbial communities were found to vary strongly across *Cephalotes* gut compartments in ways that transcend both caste and host phylogeny. Despite this, caste and host phylogeny still have detectable effects. We demonstrated microbial community divergence across gut compartments, possibly due to the various function of each gut compartment for digestion.

**IMPORTANCE** Gut compartments play an important role in structuring the microbial community within individual ants. The gut chambers of the turtle ant digestive tract differ remarkably in symbiont abundance and diversity. Furthermore, caste type explains some variation in the microbiome composition. Finally, the evolutionary history of the *Cephalotes* species structures the microbiome in our study, which elucidates a trend in which related ants maintain related microbiomes, conceivably owing to cospeciation. Amazingly, gut compartment-specific signatures of microbial diversity, relative abundance, composition, and abundance have been conserved over *Cephalotes* evolutionary history, signifying that this symbiosis has been largely stable for over 50 million years.

## Abstract (precis)

Gut compartments play an important role in structuring the microbial community within individual ants. The gut chambers of the turtle ant digestive tract differ remarkably in symbiont abundance and diversity.

## INTRODUCTION

Bacterial communities in animal guts play significant roles in animal evolution (1, 2). The diversity of eukaryotic gut bacteria is driven by host diet, distinct physiological niches across the gut habitat favoring differing microbes, and the capacity of organisms to transfer bacteria to their progeny (3–5). Bacterial gut symbionts can promote effective digestion, immunologic defense, and metabolic regulation (6).

Ants are an extremely successful clade of animals both in terms of their sheer biomass as well as their diversity, and their associated microbes play an important functional role for species of various dietary types. For example, microbial symbionts have been hypothesized to largely enable the ant’s ecological dominance throughout nutrient-limited rainforest canopies (7, 8). In addition, broad molecular surveys have illustrated that symbiotic bacteria are correlated with the evolution of herbivory across ant species (9), while both experimentation and genomics have illustrated that herbivorous ants benefit from specialized, nitrogen recycling gut bacteria (10–13).

There are striking differences between microbial communities within predatory versus herbivorous ants (14). Army ants exhibit a carnivorous diet and harbor a small number of seemingly specialized bacterial species that are common across army ant lineages (15). Herbivorous ants from Camponotini obligately use *Blochmannia* gut symbionts to nutritionally upgrade their diet with critical amino acids and possibly aid in recycling nitrogen (11). The benefits of the *Blochmannia* gut symbionts could be a reason for the evolutionary success of the Camponotini tribe (16).

In recent years, the bacterial communities of ants from the Cephalotini tribe have been studied extensively (17–21). This tribe consists of two sister genera of ants: *Cephalotes* and *Procyrptocerus*. Both genera are canopy nesters and foragers that consume extrafloral nectar, fungi, pollen, and occasionally mammal urine and bird droppings (22, 23). The diet of the adult workers is almost exclusively liquid food (24). There are 119 extant species within the turtle ant genus *Cephalotes* and 44 species of *Procryptocerus*. Across *Cephalotes*, these ants exhibit a remarkably conserved bacterial community composition. However, bacterial communities differ slightly across colonies and, seemingly, species, primarily in the changing relative abundance and presence/absence of operational taxonomic units (OTUs) from core symbiotic taxa (21). Turtle ants also demonstrate a conserved N-recycling role of their core gut symbionts. This was empirically shown in *Cephalotes varians* and through metagenomic analyses of core symbiont gene content across the genus within this same study (12). Together, these findings uncovered a nutritional mutualism between turtle ants and their gut microbes.

*Cephalotes* microbes are highly conserved within their hosts, similar to other eusocial arthropod groups (25). Various factors have been hypothesized to explain this conservation, including oral-anal trophallaxis, in which newly eclosed adult ants consume anal secretions of older adult siblings. This trophallactic behavior appears to inoculate symbiont-free, young adults with adult-enriched, specialized core symbionts (21, 26, 27). It is relatively uncommon in most ant clades but is prevalent in *Cephalotes* and *Procryptocerus* (28, 29).

After trophallactic colonization in early adulthood, specialized, adult-enriched symbionts appear to colonize multiple gut chambers, proliferating to establish large populations within adult Cephalotine ants (18, 26, 30–33). The precise composition of communities across gut regions remains obscure outside two *Cephalotes* species (18, 26). Detailing the precise trends of localization across a broader range of Cephalotine taxa will aid in our attempts to understand the evolution of ant-symbiont associations and the ways by which symbionts function to improve host ant fitness.

The basic structure of the adult digestive tract is conserved across all ants, consisting of three main subdivisions: the foregut, midgut, and hindgut. The ant foregut and hindgut are further subdivided into functionally discrete subcompartments: the esophagus and crop within the foregut and the ileum and rectum within the hindgut (34). As part of foraging, ant workers collect liquid foods, which are stored temporarily in their crop. The crop is often thought of as a “social stomach,” since the undigested food can then be regurgitated and shared among nestmates (28, 35). Due to the function of the crop in temporary food storage, it is large and often dilated within *Cephalotes*.

One reason that *Cephalotes* ants exhibit unusually high partner fidelity with their gut bacteria over evolutionary time may be their highly modified proventriculus, the valve separating the crop and midgut (36). The Cephalotine proventriculus consists of a mushroom-like nodule covered in a layer of hard cuticular fibers rather than a simple tube-like structure found in most other ant clades. It functions as a passive dam, with a rigid morphology and small opening, and provides a filtering mechanism that prevents most bacteria from entering the midgut (26, 31). *Cephalotes rohweri* filters out particles smaller than most bacteria; however, the filtering layer does not fully form until several days into adulthood (26). Therefore, it is argued that sterile callow adults gain their core microbes through oral-anal trophallaxis during the short window between eclosion and proventriculus maturation. Subsequently, *Cephalotes* ants may use their proventricular filter to maintain the integrity of their symbiotic bacterial communities, warding off the invasion of potential competitors and pathogens (26).

Immediately posterior to the proventriculus lies the midgut, which functions as the primary site of nutrient digestion and absorption in insects (3). Within *Cephalotes*, the midgut is covered by a peritrophic matrix that protects it against injury and concentrates digestive enzymes (31, 33). In addition, the peritrophic matrix compartmentalizes the midgut, which may provide bacteria protection against enzymatic lysis (33).

The ileum and the rectum comprise the remaining portions of the ant gut. These chambers play a major role in both water and nutrient absorption. Within *Cephalotes*, the ileum is the longest portion of the digestive tract, containing a large number of folds. The increased surface area allows for more efficient reabsorption while providing more room for bacterial attachment (31, 32). The rectum is the most distal subcompartment of the hindgut, used to stores feces while enabling further water reabsorption before waste exits the body (3).

Given the broad differences in physiology and host function across the digestive system and a lack of precise knowledge on how symbionts with increasingly defined functions are tied to gut regions, we aimed to comprehensively understand bacterial communities across a phylogenetically broad span of *Cephalotes* species. Through the use of 16S rRNA amplicon sequencing and real-time quantitative PCR (qPCR), we investigated symbiont community composition in the crop, midgut, ileum, and rectum. Through these approaches, and through replicated sampling across 11 *Cephalotes* species and one species of *Procryptocerus*, we inferred broad-scale patterns of Cephalotine microbiome composition and variability across gut chambers. Finally, we studied the impacts of the host phylogeny on the microbiome and the potential for microbiomes to vary across castes.

## RESULTS

A total of 492 samples were sequenced with four control samples (see Data Set S1 in the supplemental material). The 16S rRNA amplicon sequencing raw reads are available from NCBI via BioProject record SAMN16421870. There were a total of 10,380,596 reads from the raw data set. Within the rarefied data set, there were no crop samples for *C. cordatus* samples due to the very low abundance of amplicon sequence variants (ASVs) in those samples. For the rarefied data set with the *Procryptoceru*s samples included, at a sampling depth of 13,335, there were 5,334,039 reads after rarefaction with a total of 400 samples after 92 samples were removed (along with the four control samples). For the rarefied data set with the *Procryptocerus* samples excluded, at a sampling depth of 13,814 there were a total of 5,055,971 reads left after rarefaction with a total of 81 samples removed due to rarefaction (along with the four controls and 45 *Procryptocerus* samples) for a total data set of 366 samples. We excluded *Procryptocerus* samples from the main analyses to focus on the similarity and differences across digestive tissues within the *Cephalotes* samples. This data set was used when an outgroup was needed for the analyses.

### Alpha diversity metrics.

**FIG 1:**
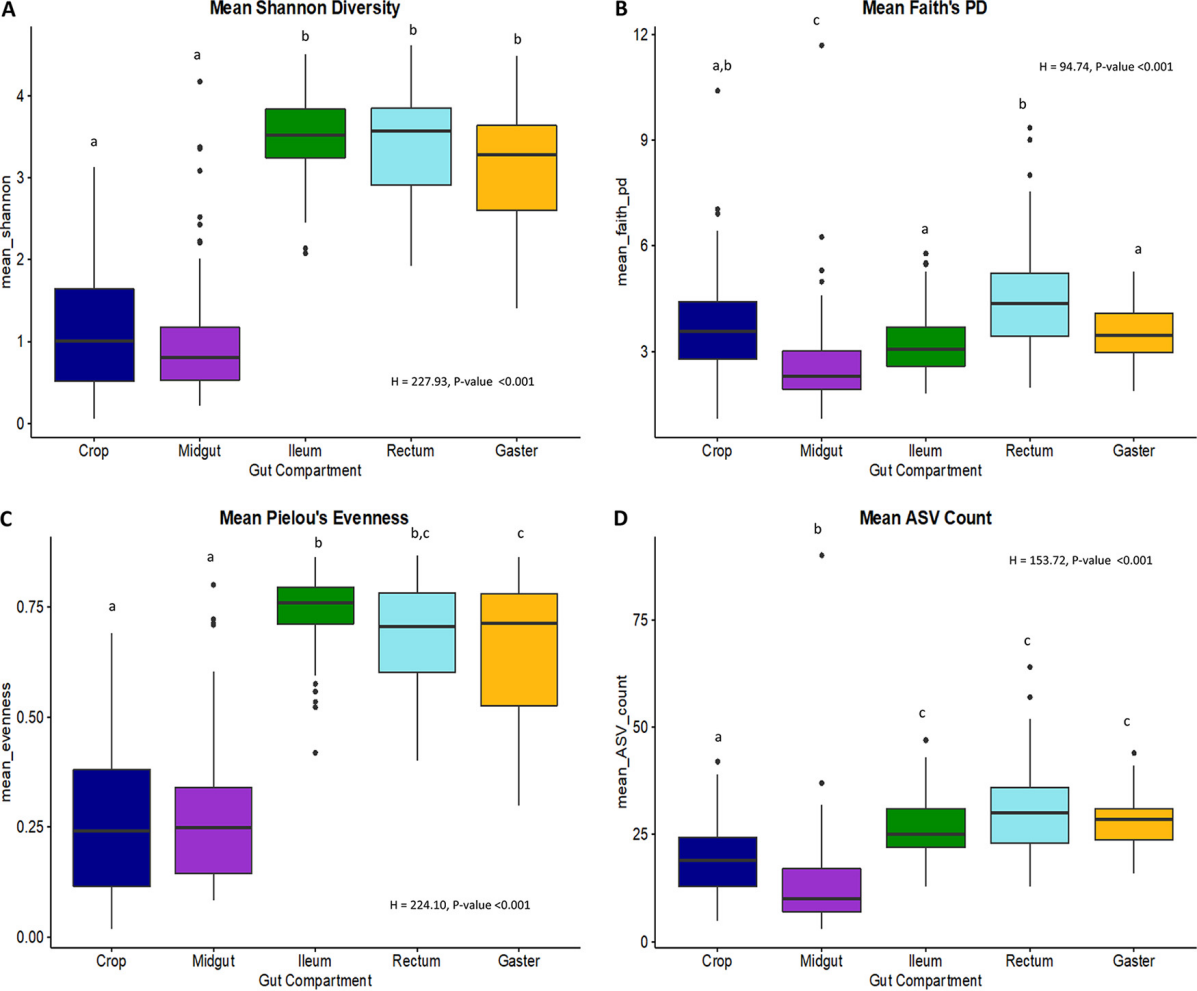
Alpha diversity metrics by body compartment, including (A) mean (± standard error) Shannon diversity, (B) mean (± standard error) Faith’s phylogenetic diversity, (C) mean (± standard error) Pielou’s evenness, and (D) mean (± standard error) ASV count. Different letters at the top illustrate body compartments with significant differences (*P* < 0.001) in this alpha diversity metric.

Across all samples, alpha diversity metrics, including Shannon diversity and Pielou’s evenness, were significantly higher in the ileum, rectum, and total gaster samples than in the midgut and crop samples (Kruskal-Wallis analysis of variance [ANOVA]; df = 4; *H* = 227.93 and *P* < 0.001 for Shannon diversity, *H* = 224.10 and *P* < 0.001 for Pielou’s evenness; corrected *P* values of <0.001 for all Kruskal-Wallis pairwise tests) (Fig. 1A and C). Crop, ileum, rectum, and gaster samples all were significantly higher in alpha diversity than the midgut samples using the Faith’s phylogenetic diversity (PD) metric as well as ASV richness (*H* = 94.74 and *P* < 0.001 for Faith’s PD and *H* = 153.72 and *P* < 0.001 for ASV richness; Fig. 1B and D). For the alpha diversity analyses of gut compartment, all caste types were combined.

No alpha diversity metric tested found soldier versus worker or soldier versus queen samples significantly different (Fig. S1A to D). Across all samples, ASV richness was significantly different based on caste type (*H* = 7.2, *P* = 0.021; Fig. S1D). ASV richness was significantly higher in worker samples than in the queen samples (*H* = 6.7 and *P* = 0.009; Fig. S1D). Shannon diversity was different (though not significantly) when grouped by caste type (*H* = 5.5 and *P* = 0.064; Fig. S1A). Specifically, Shannon diversity was significantly higher when only looking at workers compared to queens (*H* = 5.5, *P* = 0.019; Fig. S1A). Pielou’s evenness and Faith’s PD were not significantly different over all the samples (*H* = 4.38 and *P* = 0.112 for Faith’s PD and *H* = 4.14 and *P* = 0.126 for Pielou’s evenness; Fig. S1B and C). However, for Pielou’s evenness, the workers were significantly more even than the queens (*H* = 4.05, *P* = 0.044; Fig. S1C). When caste type was subdivided first by gut compartment, the only significant results were that Shannon (*H* = 2.64, *P* = 0.0247) and evenness (*H* = 2.74, *P* = 0.0184) were significantly higher in workers than in queens within the midgut and ASV richness (*H* = 2.64, *P* = 0.0252) was significantly higher in workers than in queens within the ileum. All statistics for alpha diversity metrics are found in Data Set S2.

### Bacterial abundance and quantification.

**FIG 2:**
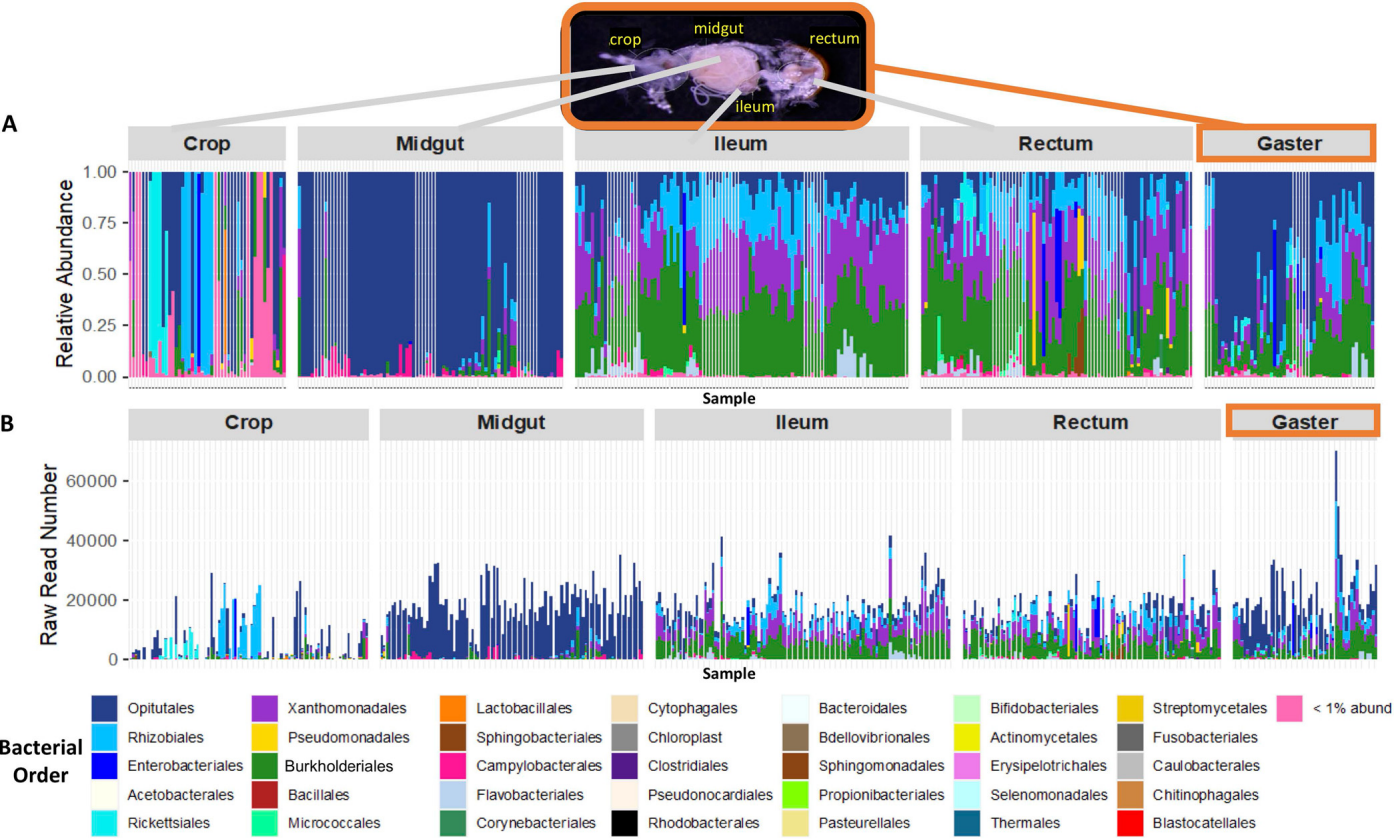
Taxon bar plots ordered by gut compartment. (A) Relative abundance plot of rarefied data set excluding *Procryptocerus* samples colored by bacterial order of ASV. The sampling depth was 13,814 reads. Each sample was a specific individual gut compartment sampled. (B) Abundance plot, raw data set excluding *Procryptocerus* samples with no rarefaction, colored by bacterial order of ASV. Each sample was a specific individual gut compartment sampled. *Cephalotes* gut image credit: Corrie Moreau.

**FIG 3:**
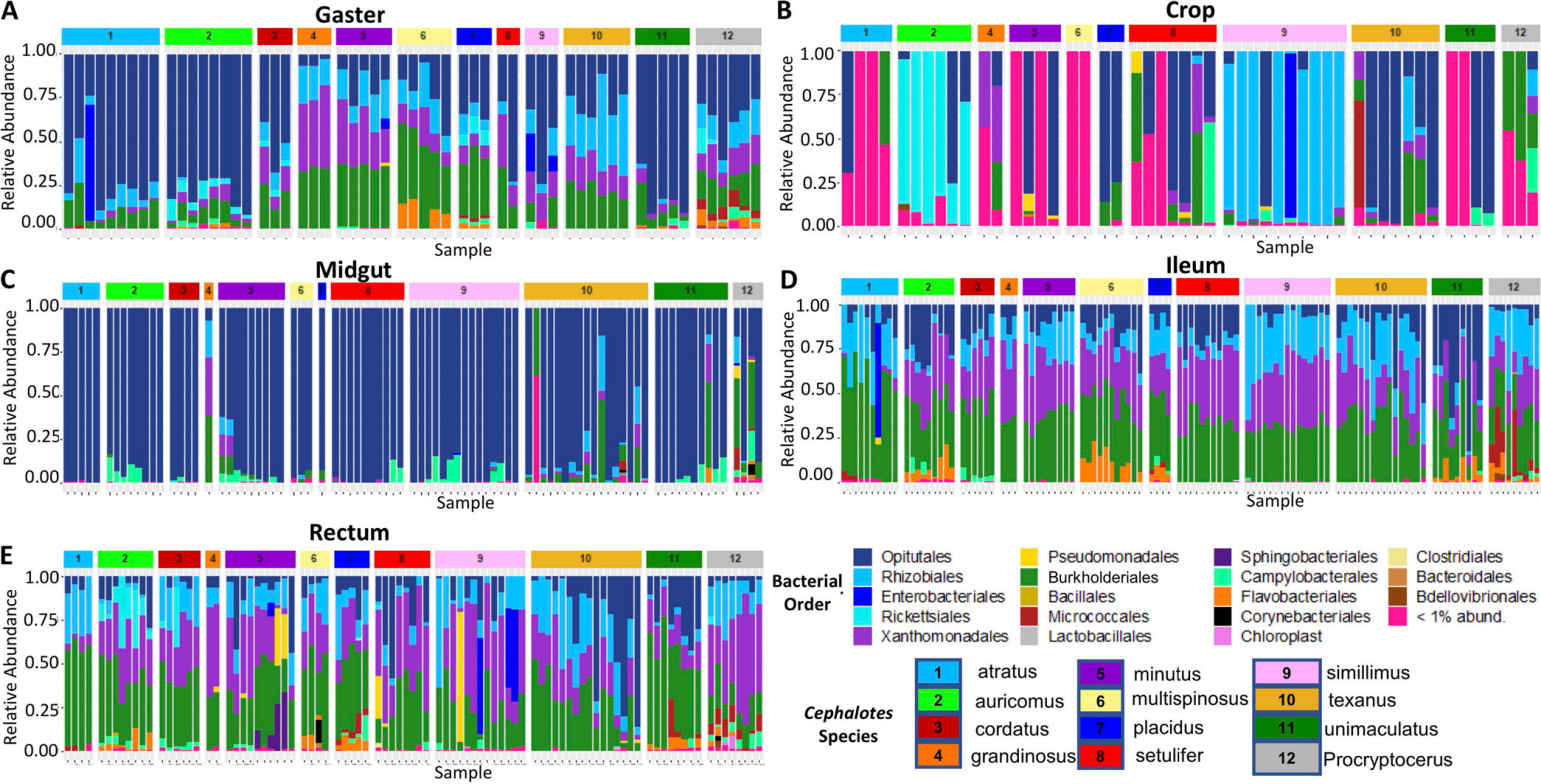
Taxon bar plot based on relative abundance ordered by *Cephalotes* species (with a number and color corresponding to species), and the relative abundance bars are colored by percentage of bacterial order of ASV. (A) Gaster samples. (B) Crop samples. (C) Midgut samples. (D) Ileum samples. (E) Rectum samples.

The top six most abundant bacterial orders overall within this 16S rRNA amplicon data set were *Burkholderiales*, *Enterobacteriales*, *Opitutales*, *Rhizobiales*, *Rickettsiales*, and *Xanthomonadales* (Fig. 2A). The relative abundance plot shown in Fig. 2A illustrates that the samples from the midgut, ileum, rectum, and gaster exhibited consistent bacterial orders across all samples within their respective gut compartments. On the other hand, the crop samples were not consistent and, on average, the largest contributors to the relative abundance of the microbial communities were *Rhizobiales*, which make up 18.6%, and *Opitutales*, which make up 16.3% of the overall bacterial community, respectively (Fig. 3B). Most crop samples of *C. auricomus* were dominated by *Rickettsiales* (60.49%), with 100% being composed of the genus *Wolbachia*. *Cephalotes simillimus* crop microbial communities were predominantly made up of *Rhizobiales* (74.05%). Crop samples from the remaining *Cephalotes* and *Procryptocerus* species exhibited no distinct pattern (Fig. 3B), harboring a range of specialized symbionts and rare, apparent transient nonspecialists. Although the majority of the crop samples had low raw read numbers, the crop samples with higher raw read numbers, and presumably higher density, were comprised of bacteria from the same orders as specialized core *Cephalotes* symbionts (*Opitutales*, *Xanthomonadales*, *Burkholderiales*, and *Rhizobiales*) (Fig. 2B).

The midgut samples were dominated by *Opitutales* (bacterial genus *Cephaloticoccus*) (Fig. 3C). Bacteria from this taxon made up 84%, on average, of the relative abundance of the microbial community within the midgut (Fig. 3C). The microbial community composition of the ileum and the rectum samples were similar (Fig. 2A), with the most abundant orders being *Burkholderiales* (26.3% ileum versus 25.7% rectum), *Xanthomonadales* (20.89% ileum versus 20.15% rectum), *Opitutales* (12.59% ileum versus 12.15% rectum), and *Rhizobiales* (12.43% ileum versus 11.6% rectum) (Fig. 3D and E). A cooccurrence Venn diagram illustrating the degree of overlap of bacterial ASVs demonstrated that bacterial ASVs differed among the four gut compartments (Fig. S2). In addition, it further indicates that the ileum and the rectum have the largest number of ASVs in common of any two gut compartments, the crop has a proportionally lower number of ASVs in common with other gut compartments, and a relatively large group of core ASVs is common to all gut compartments.

**FIG 4:**
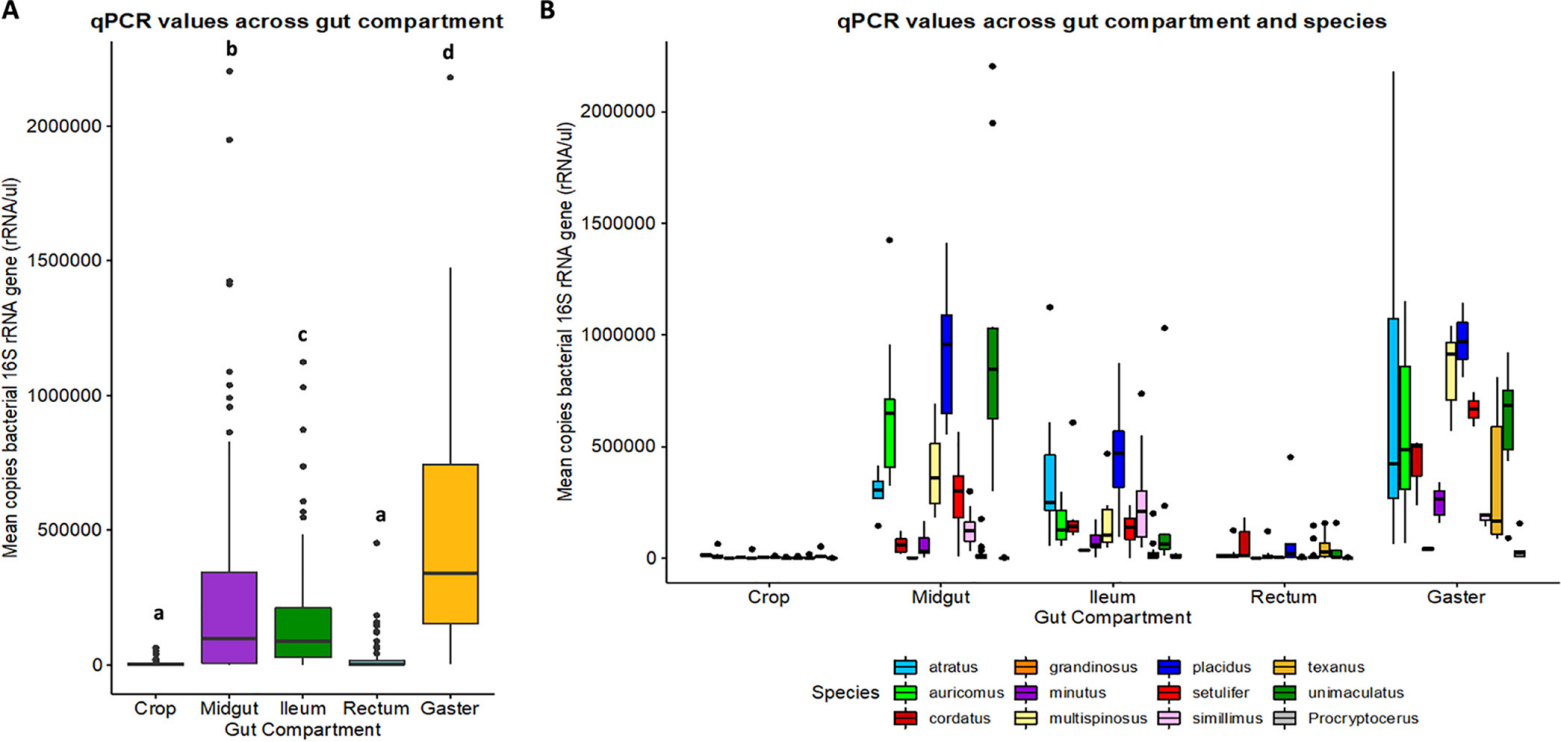
Mean qPCR values (number of copies of bacterial 16S rRNA gene [rRNA/μl]) across all samples by gut compartment (colored by gut compartment) (A) or by gut compartment and species (colored by species) (B). All gut compartments had significantly different numbers of bacteria than each other, except for the crop and the rectum, which were not statistically different. Significance was determined by a mixed-effect model with gut compartment as a fixed effect, species as a random effect, and Tukey’s HSD test for pairwise comparisons (*P* < 0.05). Different letters at the top of the specific compartment illustrate body compartments with significant differences.

Based on the qPCR results, the crop samples exhibited an average of 4,928 copies of bacterial 16S rRNA genes per sample (Fig. 4A). The midgut samples exhibited an average of 280,386 copies of bacterial 16S rRNA genes per sample (Fig. 4A). The ileum samples exhibited an average of 157,463 copies of bacterial 16S rRNA genes per sample (Fig. 4A). The rectum samples exhibited an average of 23,778 copies of bacterial 16S rRNA genes per sample (Fig. 4A). Finally, the gaster samples exhibited an average of 482,949 copies of bacterial 16S rRNA genes per sample (Fig. 4A). These results mirrored read numbers from our raw16S rRNA data (Fig. 2B).

All linear mixed models (LMMs) incorporating caste as a fixed effect had higher Akaike information criterion (AIC) values, which indicates that caste does not correlate strongly with qPCR values. The best fit LMM (lowest AIC value) incorporated gut compartment as the sole fixed effect (Table S1). Gut compartment correlated strongly with qPCR values (*P* < 0.0001) (Table S1). The mean bacterial qPCR abundances within the midgut, ileum, and gaster had significantly more bacterial abundance than the crop and the rectum (Fig. 4A). The crop and the rectum were the only gut compartments that were not statistically different from each other with regard to bacterial abundance (*P* = 0.8272) (Fig. 4A, Table S2). When these values are plotted by both species and gut compartment, it looks as though there is variation by species, but that crop and rectum have significantly lower bacterial abundance for every species sampled (Fig. 4B).

### Beta diversity metrics.

**FIG 5:**
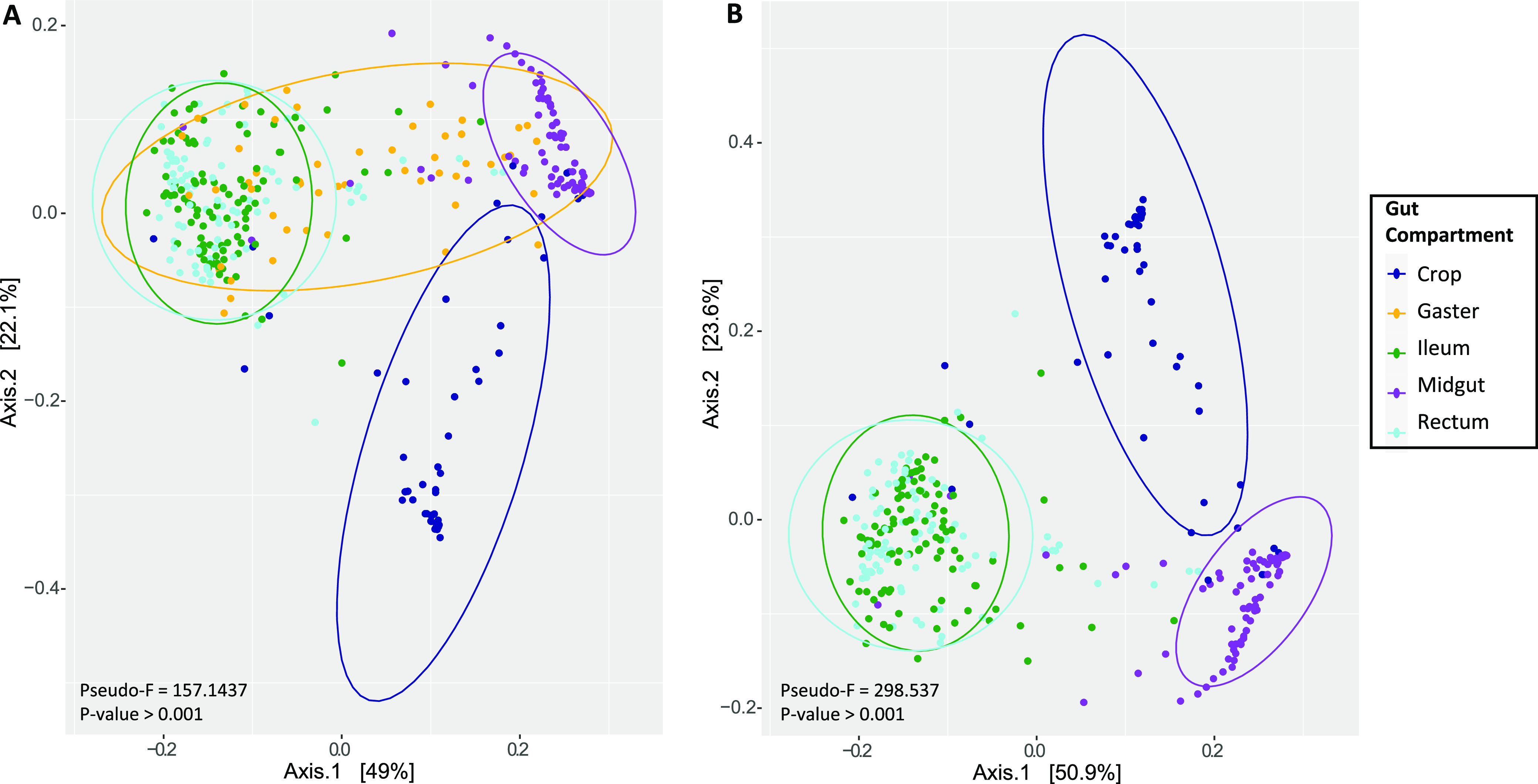
Plots of principal coordinate analysis (PCoA) with weighted UniFrac distance matrix with *Procryptocerus* samples excluded. Colors correspond to the gut compartment. (A) Whole gaster samples are included. (B) Whole gaster samples are excluded.

We performed permutational multivariate analysis of variance (PERMANOVA) on weighted UniFrac (wUniFrac) and Bray-Curtis distances calculated from the rarefied data set without *Procryptocerus* to test for dissimilarities in microbial community composition among samples based on ASVs and the variables gut compartment, species, caste, and colony (Table S3). Using a principal coordinate analysis (PCoA) for visualization, the wUniFrac distances demonstrated that the ASV samples significantly clustered by gut compartment type (pseudo-F for gut compartment, 157.143; *P* > 0.001) (Fig. 5A, Table S3). Although species, colony, and caste type all were significant, the gut compartment variable had, by far, the largest pseudo-F statistic and helps explain the clustering of the samples by gut compartment (Table S3). Whole gaster microbial community samples overlapped every other gut compartment. In addition, the ileum and rectum samples were not distinct from each other, whereas every other gut compartment was significantly distinct from one another based on pairwise PERMANOVA (Table 1). When we removed the whole gaster samples from the data set, the wUniFrac PCoA again clumped samples by gut compartment, with the ileum and rectum overlapping and the midgut and crop samples both being distinct from every other gut compartment based on their microbial community composition over all axes (pseudo-F for gut compartment, 298.537; *P* > 0.001) (Fig. 5B).

**TABLE 1:** Pairwise PERMANOVA results of different gut compartments with the Jaccard distance matrix^*a*^

X1	X2	Sample size	Pseudo-F	*P* value	pval-Bon
Crop	Gaster	100	45.746554	0.001	**0.011**
Crop	Ileum	150	128.470497	0.001	**0.011**
Crop	Midgut	129	152.16059	0.001	**0.011**
Crop	Rectum	131	90.7966672	0.001	**0.011**
Gaster	Ileum	154	22.354784	0.001	**0.011**
Gaster	Midgut	133	43.8965646	0.001	**0.011**
Gaster	Rectum	135	14.8418312	0.001	**0.011**
Ileum	Midgut	183	252.145455	0.001	**0.011**
Ileum	Rectum	185	2.19091684	0.034	0.34
Midgut	Rectum	164	166.291863	0.001	**0.011**

a Results highlighted in boldface indicate *P* values of less than 0.05. pval-Bon, Bonferroni-corrected *P* value.

**FIG 6:**
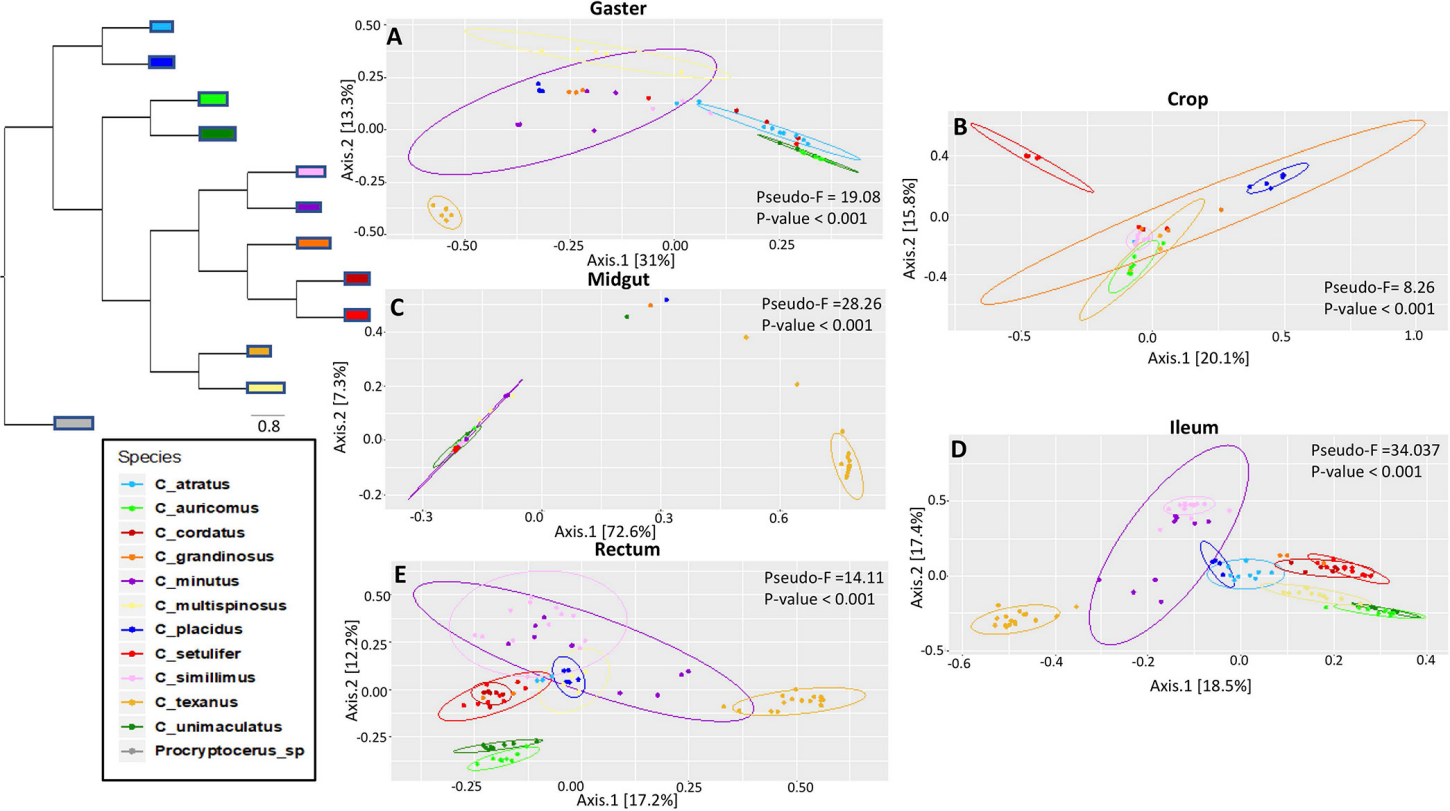
Principal coordinate analysis (PCoA) with Bray-Curtis dissimilarity distance matrix excluding *Procryptocerus* samples. *Cephalotes* phylogeny from Price et al. (85) on the left of the PCoA plots. Colors correspond to *Cephalotes* species. (A) Gaster samples. (B) Crop samples. (C) Midgut samples. (D) Ileum samples. (E) Rectum samples.

For the PCoA using the Bray-Curtis dissimilarity matrix, host species had the largest F statistic, although gut compartment, caste type, and colony variables were also significant (Fig. S3, Table S4). Visually, all of the samples clustered together except for the *C. texanus* samples, which formed a distinct cluster (Fig. S3; pseudo-F for species, 77.777; *P* > 0.001). To further examine this pattern, we analyzed the PCoA using the Bray-Curtis dissimilarity matrix for the gaster (Fig. 6A), crop (Fig. 6B), midgut (Fig. 6C), ileum (Fig. 6D), and rectum (Fig. 6E). In the midgut, ileum, rectum, and gaster plots, we found that the *C. texanus* samples cluster together away from every other *Cephalotes* species, which form a nondistinct cluster in the gaster and midgut PCoA plots (Fig. 6A and C) and a more *Cephalotes* species-specific cluster in the ileum and rectum PCoA plots (Fig. 6D and E). For the crop PCoA plot, the only samples that cluster significantly by species are *C. simillius* samples due to the dominance of *Rhizobiales* within these crop samples (Fig. 6B).

### ASV similarity percentage analyses.

Through the similarity percentage (SIMPER) analysis run in Past3, the top 10 bacterial ASVs that were important in structuring the bacterial communities within the *Cephalotes* gut compartments were identified (Table 2). Of these, two ASVs were associated with *C. texanus* samples alone. Among the *C. texanus*-enriched ASVs, *Opitutales* ASV2 was only found in *C. texanus*, exhibiting high read numbers in every individual and the highest abundance in the midgut (Data Set S3). While not exclusive to *C. texanus*, *Xanthomonadales* ASV2 was enriched in these hosts, being found only in *C. minutus* across the 11 remaining species (Data Set S3). Finally, *Opitutales* ASV1 (a sequence with just 1-bp difference versus *Opitutales* ASV2) was completely absent from *C. texanus* samples. It was, instead, found in generally high abundance within the other sampled species, although at lower relative abundance in *C. grandinosus*, *C. placidus*, and *Procryptocerus* (Data Set S3). Results of the pairwise comparisons with the Wilcoxon rank‐sum test with false discovery rate (FDR) correction for the ASVs discussed are found in Data Set S3.

**TABLE 2:** SIMPER analysis^*a*^

Taxon	Avg dissimilarity	% contribution to difference	Cumulative (%)	Mean read no. for:				
				Crop	Midgut	Ileum	Rectum	Gaster
*Opitutales* ASV1	18.07	20.26	20.26	1.23E+03	8.90E+03	1.40E+03	988	4.44E+03
*Opitutales* ASV2	5.766	6.465	26.72	911	2.12E+03	281	597	345
*Rhizobiales* ASV1	2.954	3.313	30.04	46.9	72	1.02E+03	783	512
*Xanthomonadales* ASV1	2.872	3.221	33.26	90.6	37.2	837	690	420
*Alphaproteobacteria* ASV1	2.628	2.946	36.2	2.38E+03	0.519	1.29	7.94	104
*Xanthomonadales* ASV2	2.521	2.827	39.03	50.4	94	592	660	376
*Rhizobiales* ASV2	2.247	2.52	41.55	2.11E+03	0.654	0.559	7.65	14.4
*Xanthomonadales* ASV3	1.605	1.799	43.35	20.1	3.59	353	547	110
*Rhizobiales* ASV3	1.438	1.613	44.96	69.9	84.7	297	326	285
*Xanthomonadales* ASV4	1.431	1.605	46.57	1.06E+03	0.185	0.137	0.771	291

a This includes the top 10 main ASV taxa that contribute to the observed differences in community structure.

### Codiversification analyses.

The nonparametric Mantel test comparing the gaster (entire abdomen) Jaccard similarity index distance matrix ASV table by species and the distance matrix by *Cephalotes* phylogeny illustrated a statistically significant relationship (*r* = 0.4017, *P* = 0.006703). The Mantel tests comparing the crop and the midgut ASV sample Jaccard distance matrix to the host phylogeny did not find a statistically significant relationship but a possible trend for the midgut (*r* = 0.0455, *P* = 0.43822 for crop and *r* = 0.3963, *P* = 0.053524 for midgut). On the other hand, the Mantel tests comparing the ileum and rectum ASV sample Jaccard distance matrix to the host phylogeny found a statistically significant relationship (*r* = 0.3534, *P* = 0.039836 for the ileum and *r* = 0.2485, *P* = 0.037296 for the rectum). Of the six most abundant bacterial orders, the two significantly correlated with the *Cephalotes* phylogeny were *Opitutales* (*r* = 0.4017, *P* = 0.0356) and *Burkholderiales* (*r* = 0.332, *P* = 0.00325). Results for all Mantel tests are found in Table 3.

**TABLE 3:** Mantel tests by gut compartment and bacterial lineage tested against host phylogeny from Price et al. (85)^*a*^

Subject	*r* statistic	*P* value
Overall samples	0.2452	0.11188
Crop	0.0455	0.43822
Midgut	0.3963	0.053524
Ileum	0.3534	**0.039836**
Rectum	0.2485	**0.037296**
Gaster	**0.547**	**0.006703**
*Opitutales*	0.4017	**0.035567**
*Rhizobiales*	0.1339	0.25759
*Xanthomonadales*	0.1893	0.19703
*Rickettsiales*	−0.02352	0.60958
*Enterobacteriales*	−0.1854	0.79937
*Burkholderiales*	**0.332**	**0.00325**

a Based on 9,999 permutations. Results highlighted in boldface indicate *P* values of less than 0.05. The taxon-specific tests were performed with a subset of the 16S rRNA ASV data by excluding all other bacterial orders.

Of the top six most abundant bacterial orders, *Enterobacteriales* and *Rickettsiales* were found to be negatively correlated based on the results of their Mantel tests (*r* = −0.2352, *P* = 0.61 for *Rickettsiales* and *r* = −0.1854, *P* = 0.80 for *Enterobacteriales*). Neither of these bacterial orders cospeciate or are host specific within *Cephalotes* (14, 18). *Rickettsiales* (*Wolbachia*) is often found at relatively high prevalence within *Cephalotes* species, but there is no evidence that *Rickettsiales* or *Enterobacteriales* is codiversifying due to symbiosis with *Cephalotes* species.

## DISCUSSION

Host-associated microbes perform a myriad of beneficial functions within their host. Across insects, microbes are involved in increasing tolerance to environmental perturbations (37, 38), priming the immune system (39–41), and aiding in digestion and nutrition (42–44). Within ants, gut-associated microbes contribute to a variety of nutritional adaptations (9, 11, 45). Herbivorous turtle ants maintain a remarkably conserved symbiotic bacterial community, and the constituent microbes utilize recycled waste nitrogen to synthesize amino acids (12).

Since digestive function is not uniform across the alimentary canal, we expect the microbial community to be variable across the gut compartments within *Cephalotes*. While gut compartmentalization of microbes has been well studied in mammals and birds (46–48) and has been observed in insect groups such as termites (49) and beetles (50), few studies have documented specific microbial gut compartmentalization within ants. To understand if this pattern is common across Cephalotine ants, we examined the microbial community within each gut compartment in a variety of species spanning the *Cephalotes* phylogeny.

### Gut compartment helps determine microbial community structure.

Studies of a subsocial, wood-feeding beetle, *Odontotaenius disjunctus*, found their gut contains four discrete compartments, each with distinct microbial communities (50). There is evidence that this is valuable in the aerobic and anaerobic mechanisms of energy extraction of woody material (51, 52). In addition, within both soil- and wood-feeding termites (*Cubitermes* and *Nasutitermes*, respectively), morphological differentiation of their digestive tracts creates specialized environments that aid microbial metabolic processes that permit survival on hard-to-digest food sources (49, 53). Pollen is essential to the bee diet; however, its cell wall is indigestible by the honeybee itself. Instead, bacteria found within the honey bee gut degrade pectin in the cell wall (54). The highly compartmentalized organization of bacteria within the honeybee gut may aid the bacteria in digesting recalcitrant forms of carbon from the pollen component of their diet.

**FIG 7:**
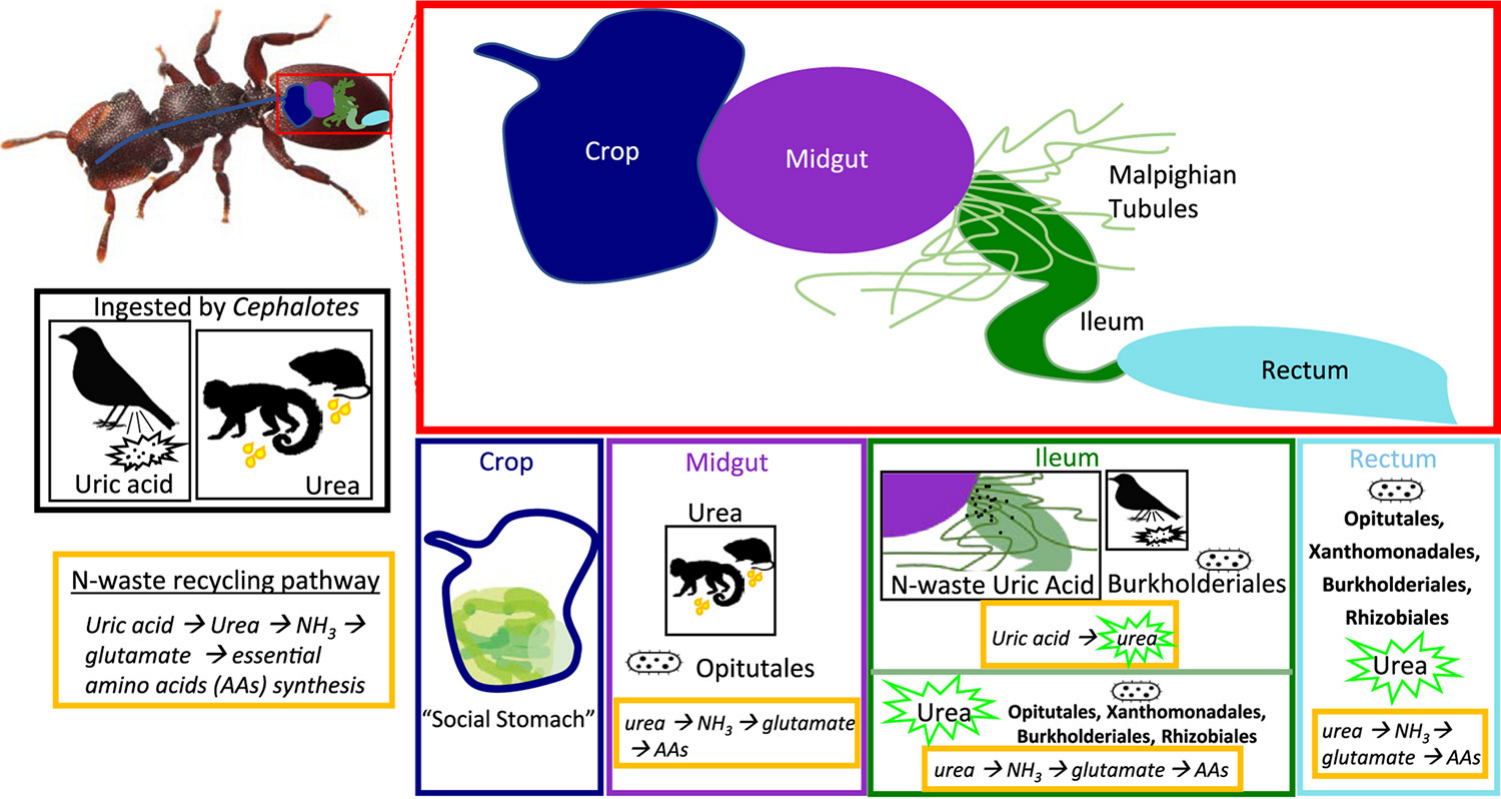
Illustration of gut compartments within the *Cephalotes* digestive tract with main bacterial symbionts and N-waste recycling pathways. The crop considered the social stomach hosts a diverse collection of transient bacteria at low abundance. The midgut contains primarily *Opitutales* bacteria that have the potential to assist in urea metabolism and amino acid biosynthesis. The uric acid found within the ileum could be degraded into urea with assistance from *Burkholderiales* bacteria, with this urea then being converted to amino acids with the aid of bacteria from the orders *Opitutales*, *Xanthomonadales*, *Rhizobiales*, and *Burkholderiales*. Compared to the ileum, the rectum contains a less abundant, though similar, suite of bacterial symbionts, possibly aiding in nitrogen recycling and amino acid biosynthesis but at a much smaller scale. (*Cephalotes* image courtesy of Steven Wang; reproduced with permission.)

The implication of gut compartmentalization with specialized microbes is that these microbes become increasingly specialized for their host, and, over evolutionary time, the host becomes increasingly reliant on these bacteria for survival. In addition, as microbes become specialized, the gut of the insect becomes increasingly partitioned for these specialized symbionts (e.g., termite guts) (49). We found that the gut compartment emerges as an important way to structure the microbial community within the *Cephalotes* digestive tract. In particular, crop and midgut microbiomes are distinct, both from each other and from the ileum/rectum (Fig. 2A and 5B; see Fig. S2 in the supplemental material). The lack of uniformity across the digestive tract raises the possibility that symbionts perform distinct functions within each gut compartment (Fig. 7).

Some insects have a crop with abundant and diverse microbes (55, 56). However, more commonly the crop contains bacteria in low abundance or is almost entirely lacking microbes, possibly due to routine evacuation (57, 58). In our study, these crop samples often hosted a different set of microbes than the rest of the compartments (Fig. 5B), although residents were found at low abundance. In general, bacterial community composition of crops varied widely across sibling ants, colonies, and species, exhibiting few consistent patterns across all samples (Fig. 2A and 3B). This suggests that these crop samples reflect a diverse suite of bacteria that are held at low density, which could be transient microbes from food sources or those acquired from oral-anal trophallaxis. In addition, qPCR values from the crop recorded uniformly negligible amounts of 16S rRNA gene copies (Fig. 4B) and the lowest number of raw reads of any gut compartment (Fig. 2B). The low quantity of bacteria within the crop samples along with a lack of bacterial uniformity is consistent with the function of the crop as the social stomach within the ant gut (35) and not a site for specialized bacterial colonization or for major nutritional/digestive function.

The microbial community of the midgut is distinct from other gut compartments and highly similar across colonies and species (Fig. 3C). Within all 11 *Cephalotes* species sampled, the midgut samples are dominated by a single bacterial order, *Opitutales*, comprised almost exclusively of the specialized core symbiont genus *Cephaloticoccus* (Fig. 2A). This result is consistent with previous studies of *C. rohweri* midgut, which was completely dominated by what appeared to be *Cephaloticoccus* (26). Accordingly, the midgut had the lowest alpha diversity of all gut compartments based on every metric of alpha diversity we measured (Fig. 1).

Urea, a major component of mammal urine, is often ingested by *Cephalotes* species (22, 23). The abundance of *Cephaloticoccus* found in the midgut, coupled with the discovery of urease-encoding capacities across this group, suggests that they assist in urea metabolism (12). These bacteria also encode glutamate dehydrogenase (*gdhA*) and have complete pathways for synthesizing most essential amino acids (12, 59). This indicates that within the midgut, *Cephaloticoccus* has the capacity to convert urea into ammonia and ammonia into glutamate and to subsequently use glutamate as an N donor in synthesizing several essential amino acids (Fig. 7). In addition, given the high abundance of *Cephaloticoccus* in the midgut, it likely has inherent properties that allow it to outcompete other bacterial groups or to uniquely thrive under the harsh midgut conditions.

The ileum contains a combination of both partially digested liquid food and nitrogenous waste. The uric acid in the ileum likely derives from two sources: ingested bird excrement and output from the Malpighian tubules (12, 32). Malpighian tubules spread into the *Cephalotes* body cavity and absorb nitrogenous waste, like uric acid, functioning as the main excretory organ of insects (3). Uric acid can be converted into urea with the aid of *Burkholderiales* bacteria (12). From there, *Opitutales*, some *Rhizobiales*, and occasionally *Xanthomonadales* or *Burkholderiales* could further break down urea into ammonia. Host bacteria could then assimilate the ammonia into glutamate, utilizing this molecule as an N donor in the synthesis of different amino acids (Fig. 7). *Burkholderiales* ASV1 and ASV2 were most similar to *Burkholderiales* strain POW0550W-166 (100% and 99.12%, respectively, identity based on BLASTn; accession number MF441555). POW0550W-166 can produce urea from allantoin, which acts as a proxy for the latter portion of the uric acid degradation pathway (12). Since *Burkholderiales* ASVs make up the largest portion of the bacterial community within the ileum (26%), *Burkholderiales* bacteria may assist in uric acid degradation within the *Cephalotes* ileum. Microscopy studies have found large aggregations of bacterial cells along the entire ileum, with an especially high bacterial load at the midgut-ileum junction, where the Malpighian tubules deposit nitrogenous waste (12, 32, 60). Hence, the localization of uric acid degraders and symbionts likely to express urease within this chamber suggests the ileum is another important site for N-recycling and amino acid metabolism.

The similarity between the bacterial community composition of the ileum and the rectum is striking. The ileum and the rectum tightly overlap and cluster together in the weighted uniFrac PCoA plot, which incorporates abundance of the ASVs as well as phylogenetic distance (Fig. 5B). Furthermore, relative abundance of reads assigned to *Burkholderiales*, *Opitutales*, *Rhizobiales*, and *Xanthomonadales* were virtually identical across these gut compartments (Fig. 2A). These similarities are not surprising, since the ileum and the rectum together form the hindgut and both function in nutrient digestion and reabsorption. This raises the possibility that N recycling and amino acid biosynthesis are further executed throughout the rectum. However, the rectum appears to harbor very small numbers of symbiotic bacteria, a finding derived from our qPCR assays and from microscopy studies of the *Cephalotes* gut (60). Thus, we propose that rectal bacteria arrive with the passage of digestate from the distal digestive tract, which may enable successful microbial transmission through oral-anal trophallaxis.

### Caste may play a role in structuring microbial communities.

In addition to gut compartment, caste may play a minor role in structuring microbial gut communities. The three distinct *Cephalotes* castes sampled were worker, soldier, and queen. While microbes within the soldier caste samples were not significantly different from those in either the worker or the queen samples, worker samples exhibited slightly higher microbial Shannon diversity, ASV counts, Faith’s PD, and evenness than the queen samples (Fig. S1). Figure S4 highlights the taxonomic composition by caste type in the data. The queen, worker, and soldier samples all appear to primarily contain microbes associated with their core symbionts (Fig. S4). This means that potentially differential caste exposure to environmental microbes most likely is not playing a major role in the structuring of the microbial gut community of any caste. In addition, there were no easily detectable differences in ASVs between workers, soldiers, and queens; therefore, more fine-scale work would help tease apart these dissimilarities.

### Evolutionary history of the host structures gut bacterial communities.

Host phylogeny comprises another correlate of bacterial community composition within the *Cephalotes* gut. This study corroborates comparative studies that find *Cephalotes*-specific microbes comprising nearly all of the gut microbiome in a variety of *Cephalotes* species (17, 18, 21, 26). The Mantel test demonstrated a significant correlation between the *Cephalotes* phylogeny and a dendrogram constructed from beta diversity measures of bacterial community similarity in the gaster (*r* = 0.547, *P* = 0.0067). This supports work from a prior study focused mostly on a different set of *Cephalotes* species, which found evidence that the observed pattern of microbial community correlation with host phylogeny was consistent with codiversification rather than diet or other selective conditions (21). In our study, among the six most abundant bacterial orders found within the ant gut, *Opitutales* and *Burkholderiales* were significantly correlated with the *Cephalotes* phylogenies. In addition, *Rhizobiales* and *Xanthomonadales* were also highly abundant but not significantly correlated with the *Cephalotes* phylogeny. All four of these bacterial orders are part of the stable core microbiome within the *Cephalotes* digestive tract and are found at high prevalence (9, 14, 17). This could provide further evidence that these core microbial groups are codiversifying within their *Cephalotes* host digestive tracts over evolutionary time.

### The influence of the host phylogeny on the bacterial community varies by gut compartment.

This study provides a finer context for understanding how the evolutionary history of *Cephalotes* is shaping the microbial community within the gut. We found that host-symbiont evolutionary correlation varied depending on the specific gut compartment within the digestive tract.

Unlike more posterior chambers, crop samples were highly variable within and between species and were not colonized by a consistent set of symbionts or environmental bacteria (Fig. 3B). Only two species provided exceptions to this overall pattern: *C. auricomus*, with crops harboring large numbers of *Rickettsiales*, and *C. simillimus*, whose crop compartments were dominated by *Rhizobiales*. In accordance with the overall trends, a Mantel test (*r* = 0.0455, *P* = 0.43822) (Table 3) suggested that host phylogeny does not correlate with community similarity of the crop microbiome. Any actual influence of host phylogeny may be hard to detect, since the crop samples with the lowest bacterial density tended to have the highest abundance of nonspecialized/core bacteria. Furthermore, a number of bacteria in the crop were not related to core symbionts, suggesting the regular occurrence of environmental microbes or an inability to remove contaminant sequences that dominated the libraries of these microbially sparse samples.

Additionally, this study did not find a discernible influence of host phylogeny on the midgut bacterial community composition, despite a possible trend (*r* = 0.3963, *P* = 0.053524). While the crop samples had low abundance and variable bacterial communities, the midgut samples had generally high bacterial abundance and were almost completely uniform in bacterial composition, i.e., dominated by a few *Cephaloticoccus* (*Opitutales*) ASVs. Thus, even though an important core symbiont dominates the *Cephalotes* midgut, the uniformity of the midgut microbiome, and our reliance on slowly evolving 16S rRNA, may obscure finer-scale impacts of phylogeny (i.e., cospeciation) detectable through alternative methods.

Within the ileum and the rectum, the host’s evolutionary history appears to influence the bacterial community composition (Table 3). These gut compartments show a relatively consistent set of bacterial orders based on the specific gut compartment and host species sampled (Fig. 3D and E). While there was some variation in how bacterial communities mirror the *Cephalotes* host phylogeny here, there was still host-specific phylogenetic clustering (Fig. 6D and E). The ileum illustrated the greatest degree of visible clustering based on host phylogeny, as every species overlapped and clustered with their most closely related sister species, with the exception of *C. texanus/C. multispinosus* (Fig. 6D). The core bacteria found in the ileum may be driving this clustering based on host phylogeny. Since the ileum is the compartment where *Burkholderiales*, *Opitutales*, *Rhizobiales*, and *Xanthomonadales* are all found at high relative abundance, they may exhibit important nutritional functions. More in-depth studies specifically examining phylogenetic congruence on the *Cephalotes* bacterial microbiome are needed to more definitively illustrate potential cospeciation based on the gut compartment.

### *Cephalotes texanus* has a partially distinct gut microbial community.

Despite overall similarity in order-level composition (Fig. 3), the microbial community of *C. texanus* was significantly distinct from all other species sampled across the midgut, ileum, rectum, and gaster according to ASV-based measures (Fig. 6A and C to E, Fig. S3). Within the gaster samples, *Procryptocerus* and *C. texanus* both clustered away from the rest of *Cephalotes* samples when including *Procryptocerus.* Since *Procryptocerus* is the most distantly related group in the data set, it is unsurprising that its bacterial community composition is distinct. However, *C. texanus* is not an early branching lineage within the *Cephalotes* genus. Within the sampled phylogenetic tree, *C. texanus* is a sister species to *C. multispinosus* (Fig. 6).

The majority of the *C. texanus* samples were live dissected from laboratory-kept colonies instead of wild caught and preserved in the field (stored in 95 to 100% ethanol at −20°C until extraction), like other analyzed samples. This could explain why they clustered away from the other *Cephalotes* species (Fig. S3). Sanders et al. (21) found that ethanol-preserved gasters recovered less *Opitutales* in 16S rRNA sequencing. However, we also included three wild-caught and preserved *C. texanus* samples stored in 95% ethanol that clustered with the laboratory-reared and live-dissected *C. texanus* samples (Fig. S3), arguing against preservation method as the driver of this pattern.

A small number of ASVs unique to *C. texanus* drive the distinctive nature of their microbiome. Based on the SIMPER analysis, three of the top 10 ASVs were associated specifically with (or lacking in) *C. texanus* (*Opitutales* ASV1 and ASV2 and *Xanthomonadales* ASV2). *Opitutales* ASV2 and *Xanthomonadales* ASV2 were found within *C. texanus*, whereas *Opitutales* ASV1 was found at high levels only within other *Cephalotes* species (Data Set S3). *Opitutales* ASV2 was ubiquitous and at high abundance within every *C. texanus* sample, with especially high read numbers in the midgut. With a more detailed comparison, we found that *Opitutales* ASV2 was 100% identical (based on BLASTn) to the cultured symbiont bacterial strain JDR108-110A-112 (accession number MF945635). This strain is positive for urea degradation via urease, as in other *Cephaloticoccus* strains studied to date, and contributes to the N-recycling pathway (12). It is not yet clear, then, if this symbiont differs functionally from other symbionts in this lineage or whether its sequence differentiation is reflective of substitutions accruing within a specialized bacterium showing little capacity for horizontal transfer.

### Gaster serves as a general proxy for digestive tract.

Often in microbial studies of ant guts, DNA is isolated and sequenced from the entire gaster (or abdomen) as a proxy for the digestive tract (15, 60, 61). We tested this assumption by including both whole gaster and specific gut compartments. We compared the relative abundance and microbial community composition of the whole gasters to those of the specific gut compartments (Fig. 3A and 6A). The microbial community composition of the gaster samples was found to overlap samples from all gut compartments: crop, midgut, rectum, and ileum (Fig. 2A). In addition, the qPCR values for the whole gasters were, on average, almost double any of the other gut compartments and, on average, close to the sum of the averages of each of the individual compartments (Fig. 4B). The high microbial abundance within the gaster and the microbial community composition overlap provides evidence that gaster samples can serve as a rough proxy for the microbial community found within the digestive tract. Since dissecting each minute gut compartment within the *Cephalotes* gut is difficult and time intensive, sampling whole gasters can be used for future studies examining gut symbionts within this genus. However, studies only sampling whole gasters would not be capable of discerning patterns of compartmentalization described in this study.

### Conclusions.

Our study sheds new light on the forces structuring the specialized, ancient microbial community within the digestive tract of *Cephalotes* turtle ants. The gut compartment plays a large role in shaping community structure within individual ants. Despite some taxonomic overlap between gut compartments, chambers of the *Cephalotes* digestive tract differ drastically in symbiont abundance, diversity, and relative abundance. Caste explains additional variation in microbiome composition, with queens harboring small differences in their microbiomes compared to those of workers. The evolutionary history of the *Cephalotes* species serves as the third, and final, factor found to shape the microbiome in our study, revealing a trend in which related ants harbor related microbiomes, quite possibly due to cospeciation. Remarkably, gut chamber-specific signatures of microbiome abundance, diversity, and composition have remained conserved across *Cephalotes* history, suggesting that this symbiosis has been fairly stable across a span of over 50 million years. Taken together, these findings are notable, since they illustrate that specific gut compartments may be modifying and structuring their microbial community at a finer scale, possibly due to the various physiological conditions in each gut chamber but conceivably due to the influence of bacterial competition and more active measures by hosts to distinctly regulate their microbiome. Further studies, including sequence isolates or metagenomic analysis, should aim to elucidate the specific functional nature of these bacterial communities within their respective gut compartments to better understand how they contribute to digestion and nutrition within *Cephalotes*.

## MATERIALS AND METHODS

Specimens were collected from the Southeastern United States and both Central and South America in 2015 to 2016. Twenty-eight ant colonies were collected from 11 species of *Cephalotes* and one species of *Procryptocerus*. Voucher specimens for all species are deposited in the biological collections of the Cornell University Insect Collection or Field Museum of Natural History. Alate (winged) queens, dealate queens, soldiers, and minor worker castes all were collected when possible.

These specimens were stored in 95 to 100% ethanol or RNAlater in the field and kept at −20°C until extraction or collected in the field and then laboratory reared and subsequently live dissected. Adult ant workers, queens, and soldiers were rinsed in 70% ethanol and then sterile deionized water before dissection (17). Under a microscope, ant digestive compartments were dissected using sterile forceps. Between each dissection, forceps were washed with a 6% bleach solution and then with sterile deionized water. The individually dissected gut compartments (crop, ileum, midgut, and rectum) or entire gasters were placed into separate sterile 1.5-ml tubes with 180 μl enzymatic lysis buffer (20 mM Tris·Cl, pH 8.0, 2 mM sodium EDTA, 1.2% Triton X-100) immediately before use, with lysozyme added to 20 mg/ml, and then crushed using sterile pestles. After grinding, samples were incubated at 37°C for 30 min and subsequently extracted with a Qiagen DNeasy blood and tissue kit using the protocol for tissue and the pretreatment for Gram-positive bacteria. After DNA extraction, 492 samples were prepped for sequencing. In addition, four negative controls were included for 16S rRNA amplification and library sequencing for a total of 496 samples.

Amplicon sequencing of the bacterial community was performed using the V4 region of the 16S rRNA using the primers 515F (5′-GTGCCAGCMGCCGCGGTAA-3′) and 806R (5′-GGACTACHVGGGTWTCTAAT-3′), described in Caporaso et al. (62), by following the Earth Microbiome Project (EMP) protocol. Paired-end 151-bp reads were then sequenced on the Illumina MiSeq platform (Illumina, Inc., San Diego, CA) at Argonne National Laboratory (Lemont, IL, USA).

Real-time quantitative PCR (qPCR) was performed to estimate the abundance of bacteria in each sample. The universal bacterial 16S rRNA primers 515F and 806R were used (62). All qPCRs were conducted in triplicate on a CFX Connect real-time system (Bio-Rad, Hercules, CA) using SsoAdvanced 2× SYBR green supermix (Bio-Rad) and 2 μl of sample DNA extraction. Standard curves were generated from serial dilutions of linearized plasmid-containing inserts of Escherichia coli 16S rRNA (63, 64). We required that all qPCR standard curves have efficiency values between 90% and 110% and *R*^2^ values above 0.9. Each sample was also quantified via the Qubit fluorometer using the Qubit double-stranded DNA high-sensitivity assay kit (Thermo Fisher, Waltham, MA). The cycle number was log transformed to yield a linear starting copy number. These log-transformed mean SQ values (starting quantity estimate for the sample) from the qPCR data were standardized by total DNA concentration as determined by Qubit fluorometry (65). We used ggplot2 (66) to plot all qPCR results in R and tested the correlation of qPCR estimates of bacterial abundance using linear mixed models (LMMs) with the lme4 package in R (67). For these LMMs, gut compartment and caste were treated as fixed effects, and colonies nested within host species were treated as random effects. The Akaike information criterion was used to select the best model. Pairwise comparisons to assess significance of differences within specific gut compartments were made with the Emmeans package in R using Tukey’s honestly significant difference (HSD) method (67).

To determine bacterial composition in each gut compartment from across species in the Cephalotini, 16S rRNA amplicon sequencing data were analyzed with the open-source QIIME2 pipeline (68–70). Sequences were quality filtered using the DADA2 algorithm (89). This algorithm joins paired-end reads and then uses a quality-aware correcting model for amplicon data, which denoises, removes chimeric sequences and residual PhiX reads, dereplicates DNA reads, and subsequently calls amplicon sequence variants (ASVs). ASVs are used as a proxy for bacterial species and are similar to OTUs (operational taxonomic units) but at a finer-scale resolution (100% similarity). Paired-end sequence reads were trimmed (both reads were trimmed at 12 and truncated at 150). An alignment was made using MAFFT (71), and an ASV phylogeny was inferred from these sequences using RAxMLv8.1.16 (72). These samples were normalized with a sampling depth of 13,335 reads. Sequences were assigned to taxonomic groups using the SILVA_132_QIIME database to train our specific classifier (73, 74). Contaminants from negative controls were filtered using the Decontam package (75).

To create a second data set that only contained *Cephalotes* species and excluded samples from the sister genus (*Procryptocerus*), all of the *Procryptocerus* samples were excluded and the data set was rarefied at a sampling depth of 13,814. This rarefied data set with *Procryptocerus* samples excluded is the one used for subsequent analyses unless noted.

Alpha diversity metrics were computed to measure the richness of the communities within samples: Shannon index and Faith’s phylogenetic diversity index, Pielou’s evenness, and ASV richness (76–79). These metrics were all computed in QIIME2 using the Kruskal-Wallis pairwise comparison test. Relative abundance plots were created using Phyloseq (80) to assess the bacterial community composition among all samples. Variables such as gut compartment and caste type were tested under these alpha diversity metrics. Caste type samples were tested as both pooled samples based on caste as well as being subdivided by gut compartment and then tested by caste type within that specific compartment.

Differences in beta diversity between samples were measured with a permutational multivariate analysis of variance (PERMANOVA) on weighted and unweighted Unifrac distance matrices as well as a Bray-Curtis dissimilarity matrix. Principal coordinate analysis ordination was calculated based on the Bray-Curtis dissimilarity matrix using Adonis (81) from the Vegan package as well as in QIIME2 (82), which performed a PERMANOVA based on Bray-Curtis and weighted UniFrac distance matrices (999 permutations). The subsequent PCoA plots were then visualized using ggplot2 (80). This was used to test for differences in beta diversity among ant species, castes, colony, and sample type, i.e., particular gut compartments or whole gasters/abdomens, containing the entire gut. The R package *ecodist* (83) was used to test for host phylogenetic signal correlating with similarities in distribution and abundance by comparing the *Cephalotes* ant phylogeny and the Jaccard distance matrix from the ASV table using the Mantel test (84). To compare to the host phylogeny, we used the ant phylogeny from Price et al. (85), with the drop.tip function in *ape* (86) to prune the phylogeny to only the 12 ant species examined in this study (Fig. 6, top left phylogeny). We used the subset_taxa function within Phyloseq to subset the rarefied data sets to only contain specific bacterial orders and subsequently test them for host phylogenetic signal using the Mantel test.

Using the Past3 program, similarity percentage analysis (SIMPER) was implemented to determine specific ASV contributions to the structure of the bacterial communities (87, 88). Subsequently, boxplots were created for what SIMPER identified as the top 10 most important ASVs contributing to the bacterial community composition within *Cephalotes* gut compartments. All boxplots were made using the plugin *ggplot2* within R, and statistical tests between groups were performed using the Wilcoxon rank‐sum test with FDR correction (*P* < 0.05) (66). The code for this study is available at github.com/peterjflynn/cephalotes_gut_localization.

## Supplementary Material

Supplemental file 1

Supplemental file 2

Supplemental file 3

Supplemental file 4
