# Supplementary material for: Localization of Bacterial Communities within Gut Compartments across Cephalotes Turtle Ants
Source: Appl Environ Microbiol. 2021 Mar 26;87(8):e02803-20. doi: 10.1128/AEM.02803-20 (PMC8091110; doi:10.1128/AEM.02803-20)
Supplement: Supplemental file 4 [file AEM.02803-20-s0004.pdf]

Table S1. Generalized linear mixed model selection

**Model**	**Variable**	**Fixed Effects**	**Random Effects**	**AIC**	***p***
**mixed.lmer1**	qPCR mean	~ Gut Compartment	Species/Colony	**12387**	<0.0001
**mixed.lmer2**	qPCR mean	~ Gut Compartment + Caste	Species/Colony	12390	0.6963
**mixed.lmer3**	qPCR mean	~ Gut Compartment + Caste + Gut Compartment:Caste	Species/Colony	12392	0.1158

Table S2. Pairwise comparisons of qPCR data to assess significance of differences within specific gut compartments were made with the Emmeans package in R using Tukey's HSD method

**Contrasts**	**Estimate**	**Standard Error**	**df**	**t.ratio**	***p***
Crop - Midgut	-290274	34659	421	-8.375	**<.0001**
Crop - Ileum	-152447	34420	423	-4.429	**<.0001**
Crop - Rectum	-7870	36036	423	-0.218	0.8272
Crop - Gaster	-491100	42007	430	-11.691	**<.0001**
Midgut - Ileum	137828	33021	423	4.174	**<.0001**
Midgut - Rectum	282404	34852	425	8.103	**<.0001**
Midgut - Gaster	-200826	40831	429	-4.918	**<.0001**
Ileum - Rectum	1.45E+05	3.44E+04	423	4.198	**<.0001**
Ileum - Gaster	-338653	40243	431	-8.415	**<.0001**
Rectum - Gaster	-483230	42013	432	-11.502	**<.0001**

Table S3. Adonis test results on Weighted Unifrac distance matrix from 16S rRNA amplicon bacterial sequencing of gut compartments of *Cephalotes*.

**Variable**	**Df**	**SumsOfSqs**	**MeanSqs**	**F.Model**	**R2**	**Pr(>F)**
Gut Compartment	4	6.114237	1.528559	157.1437	0.472948	0.001
Species	10	1.636126	0.163612	16.82022	0.126557	0.001
Caste Type	2	0.056639	0.028319	2.911418	0.004381	0.007
Colony	29	0.705792	0.024337	2.502038	0.054594	0.001
Gut Compartment:Species	38	1.648595	0.043384	4.460107	0.127522	0.001
Gut Compartment:Caste Type	6	0.044463	0.007410	0.761840	0.003439	0.753
Species:Caste Type	5	0.041080	0.008216	0.844661	0.003177	0.653
Gut Compartment:Colony	40	0.460156	0.011503	1.182660	0.035594	0.125
Caste Type:Colony	2	0.030145	0.015072	1.549574	0.002331	0.144
Gut Compartment:Species:Caste Type	10	0.072487	0.007248	0.745205	0.005607	0.835
Gut Compartment:Caste Type:Colony	5	0.036584	0.007316	0.75222	0.002829	0.74
Residuals	214	2.081608	0.009727	-	0.16101	-
Total	365	12.927917	-	-	1	-

Table S4. Adonis test results on Bray-Curtis distance matrix from 16S rRNA amplicon bacterial sequencing of gut compartments of *Cephalotes* species.

**Variable**	**Df**	**SumsOfSqs**	**MeanSqs**	**F.Model**	**R2**	**Pr(>F)**
Gut Compartment	4	20.6552	5.16380	70.19453	0.14295	0.001
Species	10	57.21605	5.721605	77.77705	0.395983	0.001
Caste Type	2	0.76705	0.38352	5.213477	0.005308	0.001
Colony	29	9.32935	0.321702	4.373079	0.064566	0.001
Gut Compartment:Species	38	32.45829	0.854165	11.61116	0.224638	0.001
Gut Compartment:Caste Type	6	0.9464	0.157746	2.144339	0.006550	0.001
Species:Caste_Type	5	0.474678	0.094935	1.290516	0.003285	0.073
Gut Compartment:Colony	40	5.352201	0.133805	1.818888	0.037041	0.001
Caste Type:Colony	2	0.263384	0.131692	1.79016	0.001822	0.011
Gut Compartment:Species:Caste Type	10	0.728596	0.072859	0.99042	0.00504	0.479
Gut Compartment:Caste Type:Colony	5	0.557112	0.111422	1.514629	0.003855	0.004
Residuals	214	15.74273	0.073564	-	0.108952	-
Total	365	144.4911	NA	-	1	-

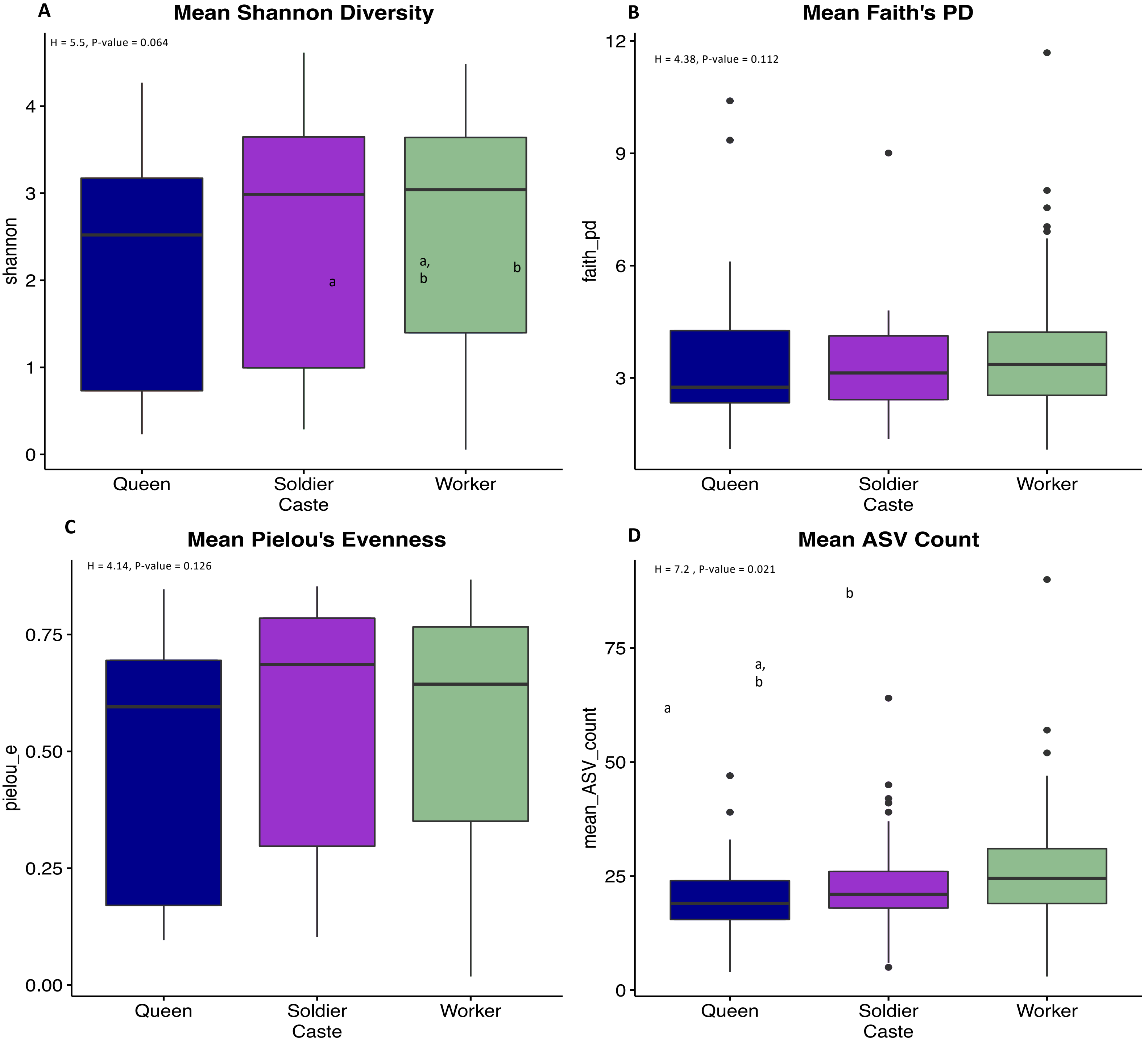

Supplemental Figure 1. Alpha Diversity Metrics by caste type including (**A**) mean (±standard error) Shannon diversity, (**B**) mean (±standard error) Faith's phylogenetic diversity, (**C**) mean (±standard error) Pielou's evenness, (**D**) mean (±standard error) ASV count. Different letters at the top of the figure illustrate body compartments with significant differences ($P<0.01$) in this alpha diversity metric.

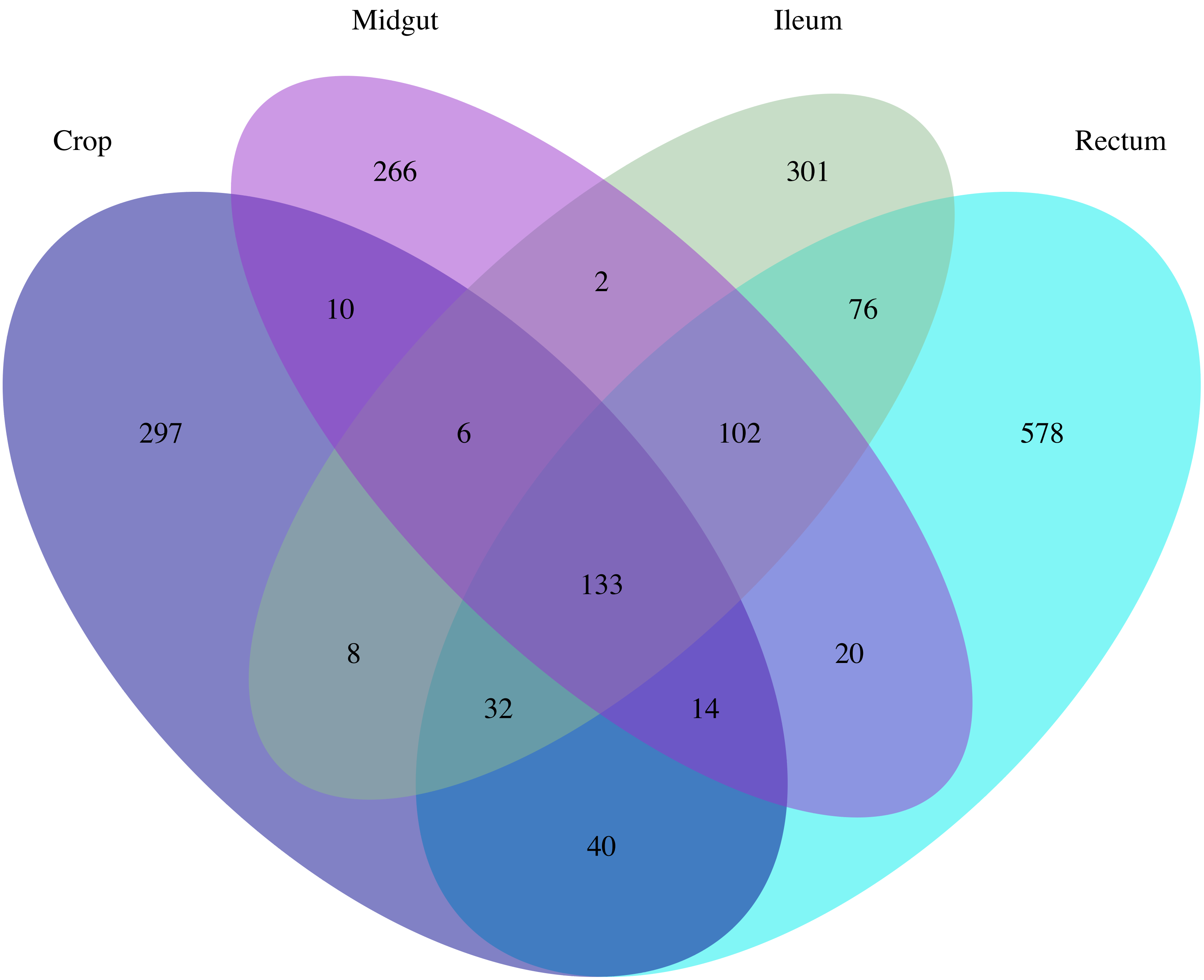

Supplemental Figure 2. Venn diagram displaying the degree of overlap and co-occurrence of bacterial ASVs found among each gut compartment.

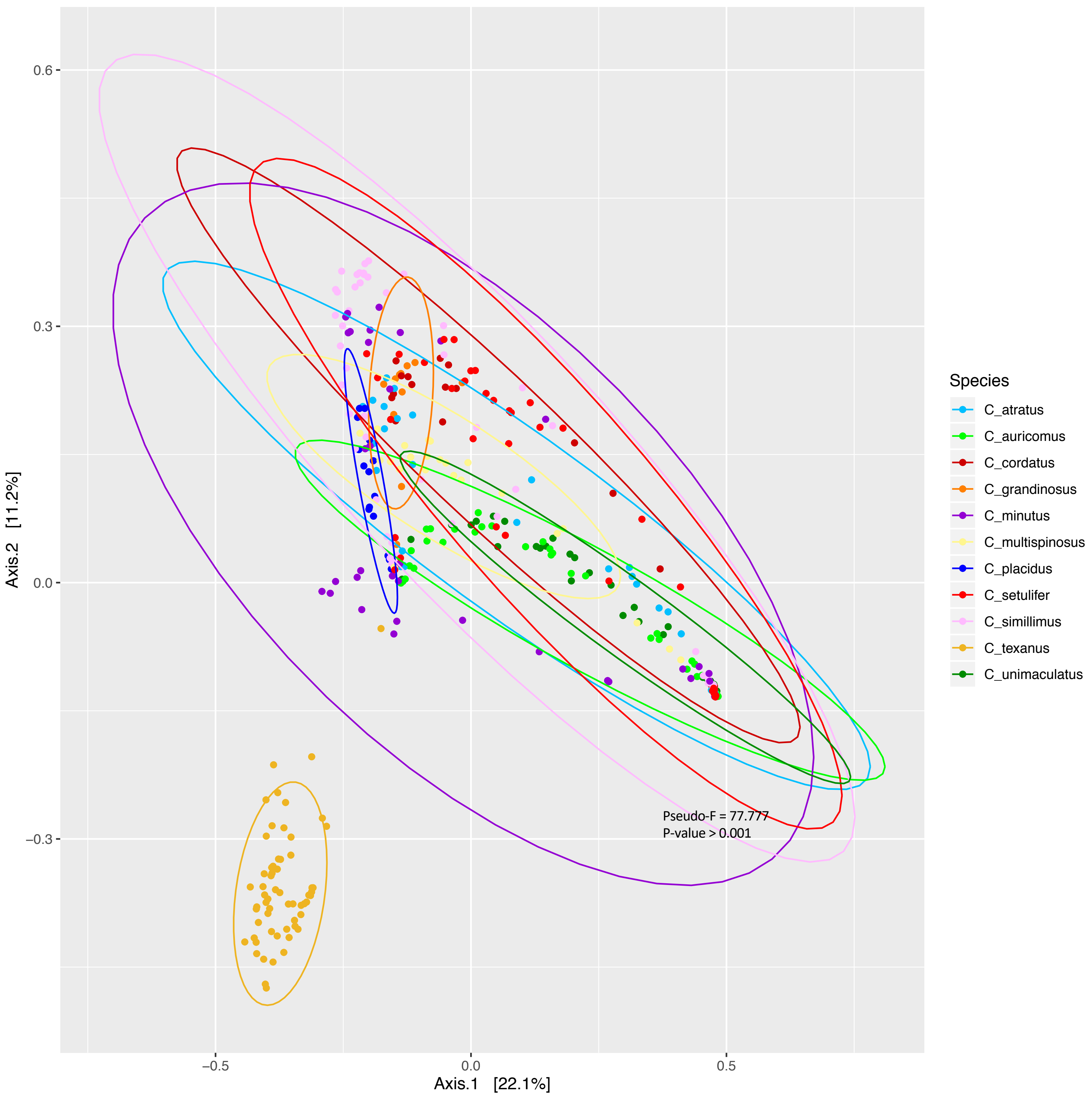

Supplemental Figure 3. Principal coordinates analysis (PCoA) with Bray-Curtis dissimilarity distance matrix. Colors correspond to Cephalotine species.

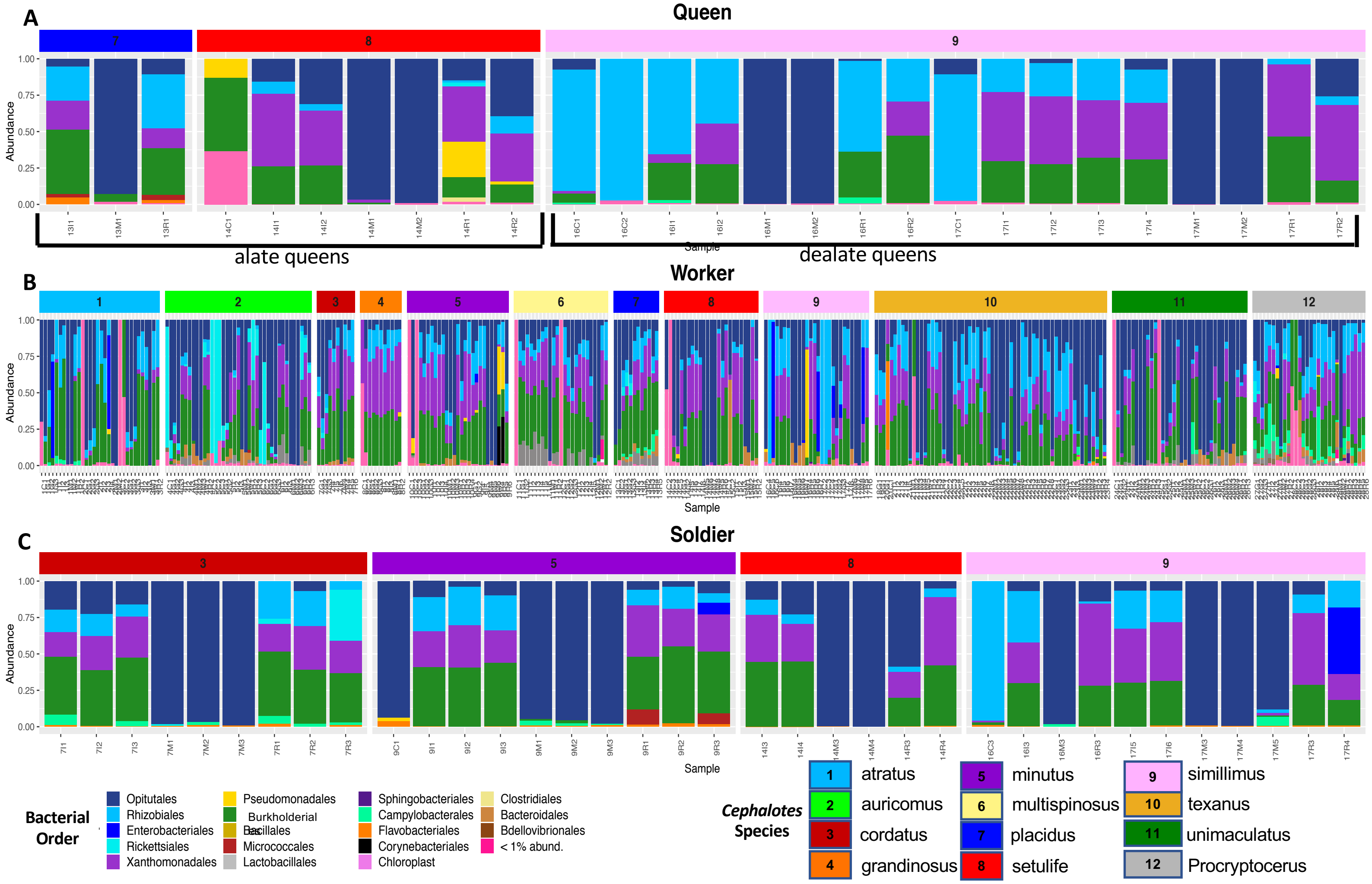

Supplemental Figure 4. Taxa Bar Plot based on Relative Abundance ordered by *Cephalotes* species (with a number and color corresponding to species) and the relative abundance bars are colored by percentage of bacterial order of ASV: (**A**) Queen caste samples (**B**) Worker caste samples (**C**) Soldier caste samples. Sample names are coded by colony number, gut section, sample replicate number (i.e. 17M3 would be ant colony 17, midgut sample, replicate 3).
